# Health Service Use and Costs During Pregnancy Among Privately Insured Individuals With Congenital Heart Disease

**DOI:** 10.1001/jamanetworkopen.2024.10763

**Published:** 2024-05-13

**Authors:** Anushree Agarwal, Rong Duan, Nasim C. Sobhani, Aarthi Sabanayagam, Gregory M. Marcus, Michelle Gurvitz

**Affiliations:** 1 Division of Cardiology, Department of Medicine, University of California, San Francisco; 2 Division of Maternal-Fetal Medicine, University of California, San Francisco; 3 Department of Cardiology, Boston Adult Congenital Heart Service, Boston Children’s Hospital, Brigham and Women’s Hospital, Harvard Medical School, Boston, Massachusetts

## Abstract

**Importance:**

Individuals with congenital heart disease (CHD) are increasingly reaching childbearing age, are more prone to adverse pregnancy events, and uncommonly undergo recommended cardiac evaluations. Data to better understand resource allocation and financial planning are lacking.

**Objective:**

To examine health care use and costs for patients with CHD during pregnancy.

**Design, Setting, and Participants:**

This retrospective cohort study was performed from January 1, 2010, to December 31, 2016, using Merative MarketScan commercial insurance data. Participants included patients with CHD and those without CHD matched 1:1 by age, sex, and insurance enrollment year. Pregnancy claims were identified for all participants. Data were analyzed from September 2022 to March 2024.

**Exposures:**

Baseline characteristics (age, US region, delivery year, insurance type) and pregnancy-related events (obstetric, cardiac, and noncardiac conditions; birth outcomes; and cesarean delivery).

**Main Outcomes and Measures:**

Health service use (outpatient physician, nonphysician, emergency department, prescription drugs, and admissions) and costs (total and out-of-pocket costs adjusted for inflation to represent 2024 US dollars).

**Results:**

A total of 11 703 pregnancies (mean [SD] maternal age, 31.5 [5.4] years) were studied, with 2267 pregnancies in 1785 patients with CHD (492 pregnancies in patients with severe CHD and 1775 in patients with nonsevere CHD) and 9436 pregnancies in 7720 patients without CHD. Compared with patients without CHD, pregnancies in patients with CHD were associated with significantly higher health care use (standardized mean difference [SMD] range, 0.16-1.46) and cost (SMD range, 0.14-0.55) except for out-of-pocket inpatient and ED costs. After adjustment for covariates, having CHD was independently associated with higher total (adjusted cost ratio, 1.70; 95% CI, 1.57-1.84) and out-of-pocket (adjusted cost ratio, 1.40; 95% CI, 1.22-1.58) costs. The adjusted mean total costs per pregnancy were $15 971 (95% CI, $15 480-$16 461) for patients without CHD, $24 290 (95% CI, $22 773-$25 806) for patients with any CHD, $26 308 (95% CI, $22 788-$29 828) for patients with severe CHD, and $23 750 (95% CI, $22 110-$25 390) for patients with nonsevere CHD. Patients with vs without CHD incurred $8319 and $700 higher total and out-of-pocket costs per pregnancy, respectively.

**Conclusions and Relevance:**

This study provides novel, clinically relevant estimates for the cardio-obstetric team, patients with CHD, payers, and policymakers regarding health care and financial planning. These estimates can be used to carefully plan for and advocate for the comprehensive resources needed to care for patients with CHD.

## Key Points

**Question:**

What are the health care use and costs among patients with congenital heart disease (CHD) during pregnancy?

**Findings:**

In this cohort study of 11 703 pregnancies, 2267 pregnancies in 1785 patients with CHD incurred $8319 and $700 higher total and out-of-pocket costs per pregnancy, respectively, than 9436 pregnancies in 7720 patients without CHD. For patients with CHD, pregnancy-related medical conditions and birth outcomes, as opposed to CHD anatomic severity, were associated with longer length of stay and higher costs.

**Meaning:**

These population-based estimates provide novel and critical data for financial planning and advocacy for adequate resources and workforce to improve care for the increasing population of patients with CHD reaching childbearing age.

## Teaser

This cohort study of US patients who are pregnant examines factors associated with health care use and costs during pregnancy in those with vs without congenital heart disease.

## Introduction

With improvements in pediatric congenital heart disease (CHD) care, the number of patients with CHD of childbearing age is increasing rapidly.^1,2^ Given the increased demands of pregnancy on cardiac physiology, these patients have increased prevalence of adverse cardiac and obstetric conditions during pregnancy.^3,4^ Despite this, only one-quarter of patients will receive American Heart Association–recommended preconception health care in the year before conception.^5^ Thus, it is important to build adequate resources and workforce to care for this growing population.

Little is known about the rates and types of health service use (outpatient and inpatient) and associated costs for patients with CHD during their pregnancy. Although there are data showing an increase in the number of hospitalizations for deliveries in patients with CHD during the past decades,^6,7,8^ these studies are limited to inpatients only and do not adequately reflect the breadth of outpatient and inpatient services used. Moreover, there are no data on the financial burden on patients (eg, out-of-pocket [OOP] costs) or payers (eg, health service costs) during pregnancy. This lack of data limits the ability to provide estimates of costs in a population-based setting to understand how comprehensive care models could be structured to create efficient workflows and improve outcomes. Therefore, in this study of a geographically representative cohort of commercially insured patients with CHD, we examine factors associated with health care use and costs during pregnancy.

## Methods

The University of California, San Francisco Institutional Review Board approved this study and determined that this research was exempt because of the use of secondary deidentified data from regulatory requirements. Patient informed consent was not required owing to the use of deidentified patient data. We followed the Strengthening the Reporting of Observational Studies in Epidemiology (STROBE) reporting guidelines.

### Setting and Data Source

The Merative MarketScan commercial database is a claims database with enrollment data from large employers and health plans across the US that provides private health care coverage for more than 273 million employees, their spouses, and dependents. The database includes a variety of fee-for-service plans, preferred provider organizations, and capitated health plans. Data about individual patients are integrated from all care settings (eg, inpatient, outpatient, and outpatient pharmacy), maintaining health care use and cost record connections at the patient level. MarketScan reports the payments that came on the paid claims submitted by various insurance carriers and provides data about total payments (total costs to all payers) and payments made by patients (OOP costs) for their coinsurance, copayment, and/or deductible.^9,10,11^ We identified enrollees aged 18 to 45 years with at least 1 full year of continuous enrollment from January 1, 2010, to December 31, 2016.

### CHD Algorithm

We identified patients with CHD if the previously published and validated *International Classification of Diseases, Ninth Revision* (*ICD-9*) and *International Statistical Classification of Diseases and Related Health Problems, Tenth Revision* (*ICD-10*) diagnosis codes for CHD were present on 2 or more outpatient claims separated by more than 30 days or 1 or more inpatient claims at any billing position during the enrollment period (eTable 1 in Supplement 1).^12,13,14,15^ For patients with codes for more than 1 CHD diagnosis, we used a hierarchical algorithm (eAppendix 1 in Supplement 1) to designate 1 condition per patient as their principal CHD diagnosis.^15^ We excluded patients with nonspecific diagnosis codes (atrial septal defect, unspecified congenital anomalies, and congenital heart block)^14,15^ and with codes documented only during pregnancy time range (to avoid inclusion of pregnant patients with fetuses affected by CHD). The remaining patients were categorized based on anatomic severity as having severe or nonsevere CHD. Randomly selected patients without CHD were matched 1:1 to patients with CHD within categories jointly defined by age, sex, and full calendar years of enrollment between 2010 and 2016.

### Pregnancy Algorithm

We applied the algorithm by Ailes et al^16^ (eAppendix 2 in Supplement 1) and used pregnancy-related procedure and diagnostic codes (eTable 2 in Supplement 1) indicating end of a pregnancy to assign birth outcome and estimate gestational age (GA) at end of pregnancy. We used estimated GA to calculate date of last menstrual period. Because Ailes et al^16^ primarily used *ICD-9* codes, we used the corresponding *ICD-10* codes from other validated studies.^17,18^ We only included participants who were completely enrolled in a contributing commercial insurance plan from 60 days before last menstrual period through 90 days after delivery. To differentiate visits associated with separate pregnancies, we required 2 or more months between 1 birth outcome and the last menstrual period of the next pregnancy. In case of any overlap between 2 pregnancies, the associated visits were included in the previous pregnancy.

### Outcome Measures

The outcomes measured for each pregnancy included health service use and costs. Health service use was categorized as follows: (1) outpatient physician visits and nonphysician encounters (eg, diagnostic testing, physical therapy, and nurse practitioner visits); (2) pharmacy claims; (3) emergency department (ED) visits (without inpatient stay); (4) inpatient visits; and (5) length of stay (LOS) while an inpatient. The outpatient physician visits were categorized as obstetrician, cardiologist, and other physician visits (primary care, neurologist, endocrinologist, gastroenterologist, heme-oncologist, infectious disease, nephrologist, pulmonologist, rheumatologist, gynecologist, or psychiatrist). These other physicians were chosen because patients with CHD often need comanagement with 1 or more of them.^2,12^

Costs included combined total payer and patient payments incurred during pregnancy and costs by setting (physician, nonphysician, pharmacy, ED, and inpatient). We separately examined the OOP costs that included patient copayments, deductibles, and payments for services not covered by insurance. We used the medical care index of the Consumer Price Index to adjust the costs for January 2024 US dollars.

### Covariates

Baseline characteristics included age, delivery year, and insurance plan at the time of delivery and the US region where the patient had the longest enrollment data. Race and ethnicity data were not reported because they were not included within the database. We categorized insurance plans as follows: (1) flexible: basic, major medical, comprehensive, or point of service with and without capitation; preferred provider organization; and consumer directed health plan; (2) network based: health maintenance organization and exclusive provider organization; (3) high-deductible health plan; and (4) unknown.

Pregnancy-related events included obstetric, cardiac, and noncardiac conditions (eTable 3 in Supplement 1); birth outcomes; and cesarean delivery. We used the codes and algorithms described previously to identify obstetric conditions (gestational diabetes, hypertensive disorders of pregnancy, and preterm premature rupture of membranes [PPROM] or preterm labor or delivery)^3,18,19,20,21^ for patients whose pregnancy lasted for more than 20 weeks of gestation (eAppendix 3 in Supplement 1). We used previously published criteria or Elixhauser comorbidity codes or single-level clinical classification system to determine cardiac (eg, heart failure, arrhythmias, coronary artery disease, coronary dissection, and peripartum cardiomyopathy) and noncardiac (eg, stroke, coagulopathy, anemia, liver disease, chronic pulmonary disease, deep vein thrombosis or pulmonary embolism, infective endocarditis, and seizure) conditions known to be associated with pregnancy.^3,22,23^ We identified all cardiac and noncardiac conditions if they were documented during the pregnancy time range but did not assess whether any of them were first diagnosed during pregnancy. All conditions were identified by 1 or more inpatient diagnosis codes or 2 or more outpatient diagnosis codes separated by 1 or more days, except for deep vein thrombosis or pulmonary embolism, that required an anticoagulation prescription claim.^21^ Birth outcomes were categorized as abortion before 20 weeks of gestation (including spontaneous pregnancy loss or termination of pregnancy), stillbirth, or live birth.

### Statistical Analysis

Data were analyzed between September 2022 and March 2024. We presented continuous variables as median (IQR) or mean (SD) and categorical variables as number (percentages). We used the χ^2^ test, 2-tailed unpaired *t* test, or Wilcoxon rank sum test as appropriate. We calculated standardized mean differences (SMDs) for service use and cost estimates. We used negative binomial mixed models^24^ to estimate mean total and OOP cost, after adjusting for all the covariates and for any significant interactions among the covariates. The negative binomial distribution accommodated for the right skewing of cost and the overdispersion characteristic of the cost. The random effect in the mixed model accounted for the correlation of multiple pregnancies of the same patient. The significance of the random effect was tested by the likelihood ratio test for the variance. We separately estimated adjusted mean cost by setting and represent the total adjusted mean cost estimate as the sum of these costs. For patients with CHD, we examined association between covariates and CHD severity and ED visits, LOS, and total and OOP cost using negative binomial mixed models. We conducted a subanalysis for live-birth pregnancies. All analysis was performed using SAS software, version 9.4 (SAS Institute Inc). A 2-sided *P* < .05 was considered significant.

## Results

### Study Cohort Characteristics

A total of 11 703 pregnancies (mean [SD] maternal age, 31.5 [5.4] years) were studied, including 1785 patients with CHD contributing 2267 pregnancies (492 pregnancies among 387 patients with severe CHD and 1775 pregnancies among 1398 patients with nonsevere CHD) and 7720 patients without CHD contributing 9436 pregnancies (eFigure 1 in Supplement 1). Baseline characteristics and pregnancy-related events of the study cohorts are given in Table 1. Compared with those without CHD, pregnancies in patients with CHD were more likely to represent Northeast and West regions and have many obstetric, cardiac, and noncardiac conditions; abortions; and cesarean deliveries but less likely to have high-deductible health plans and live births. Compared with nonsevere CHD, pregnancies in patients with severe CHD were more likely to represent Northeast and South regions and have PPROM or preterm labor or delivery and cardiac conditions.

**Table 1.** Baseline Characteristics and Pregnancy-Related Events^a^

Characteristic	All pregnancies (N = 11 703)	Pregnancies in patients without CHD (n = 9436)	Pregnancies in patients with CHD			*P* value^b^		
			Any CHD (n = 2267)	Severe CHD (n = 492)	Nonsevere CHD (n = 1775)	CHD vs No CHD	Severe vs Nonsevere CHD	Nonsevere CHD vs no CHD
Baseline characteristics								
Age at delivery, mean (SD), y	31.5 (5.4)	31.5 (5.3)	31.4 (5.7)	30.3 (5.6)	31.7 (5.7)	.26	<.001	.29
Region of residence^c^								
Northeast	2298 (19.7)	1764 (18.8)	534 (23.7)	129 (26.3)	405 (22.9)	<.001	.03	<.001
North Central	2577 (22.1)	2095 (22.3)	482 (21.4)	95 (19.3)	387 (21.9)			
South	4198 (36.1)	3537 (37.7)	661 (29.3)	160 (32.6)	501 (28.4)			
West	2566 (22.0)	1987 (21.2)	579 (25.7)	107 (21.8)	472 (26.7)			
Year of delivery								
2010-2012	5055 (43.2)	4089 (43.3)	966 (42.6)	212 (43.1)	754 (42.5)	.76	.83	.79
2013-2014	4070 (34.8)	3280 (34.8)	790(34.8)	166 (33.7)	624 (35.2)			
2015-2016	2578 (22.0)	2067 (21.9)	511 (22.5)	114 (23.2)	397 (22.4)			
Insurance types								
Flexible plans	9027 (77.1)	7242 (76.7)	1785 (78.7)	383 (77.8)	1402 (79.0)	.006	.74	.009
Network-based plans	1716 (14.7)	1402 (14.9)	314 (13.9)	67 (13.6)	247 (13.9)			
High-deductible health plans	604 (5.2)	515 (5.5)	89 (3.9)	23 (4.7)	66 (3.7)			
Unknown	356 (3.0)	277 (2.9)	79 (3.5)	19 (3.9)	60 (3.4)			
Pregnancy-related events								
Obstetric conditions, No.^d^	9052	7375	1677	357	1320			
Gestational diabetes	781 (8.6)	640 (8.7)	141 (8.4)	37 (10.4)	104 (7.9)	.72	.13	.34
Hypertensive disorders of pregnancy	969 (10.7)	742 (10.1)	227 (13.5)	46 (12.9)	181 (13.7)	<.001	.69	<.001
PPROM or preterm labor or delivery	1383 (15.3)	1070 (14.5)	313 (18.7)	85 (23.8)	228 (17.3)	<.001	.005	.009
Cardiac conditions	472 (4.0)	123 (1.3)	349 (15.4)	126 (25.6)	223 (12.6)	<.001	<.001	<.001
Noncardiac conditions	1041 (8.9)	697 (7.4)	344 (15.2)	86 (17.5)	258 (14.5)	<.001	.11	<.001
Birth outcomes								
Abortion (<20 wk)	2651 (22.7)	2061 (21.8)	590 (26.0)	135 (27.4)	455 (25.6)	<.001	.42	<.001
Stillbirth	130 (1.1)	100 (1.1)	30 (1.3)	9 (1.8)	21 (1.2)	.28	.27	.64
Live birth	8924 (76.3)	7275 (77.1)	1649 (72.7)	348 (70.7)	1301 (73.3)	<.001	.26	<.001
Cesarean delivery^d^	3319 (36.7)	2585 (35.1)	734 (43.8)	166 (46.5)	568 (43.0)	<.001	.24	<.001

Abbreviations: CHD, congenital heart disease; PPROM, preterm premature rupture of membranes.

^a^ Data are presented as number (percentage) of patients unless otherwise indicated.

^b^ *P* values were obtained using the χ^2^ test or 2-tailed, unpaired *t* test as appropriate.

^c^ Sixty-four pregnancies were missing region of residence.

^d^ For pregnancies lasting more than 20 gestational weeks.

### Patterns of Health Care Use and Costs

Compared with patients without CHD, pregnancies in patients with CHD had significantly higher health care use in all categories (SMDs ranging from 0.16 [*P* < .001] for outpatient obstetric visits to 1.46 [*P* < .001] for outpatient cardiologist visits) and significantly higher cost in all categories (SMDs ranging from 0.14 [*P* < .001] for pharmacy costs to 0.55 [*P* < .001] for outpatient physician costs) except for OOP inpatient and ED costs (Table 2). Compared with nonsevere CHD, pregnancies in patients with severe CHD had significantly higher outpatient cardiology visits, lower outpatient nonphysician encounters, and higher costs (except for similar pharmacy and ED costs), whereas OOP costs were not significantly different except for lower inpatient costs (Table 2).

**Table 2.** Health Service Use and Costs

Variable	Pregnancies in patients without CHD (n = 9438)	Pregnancies in patients with CHD			Standardized mean difference					
		Any CHD (n = 6385)	Severe CHD (n = 672)	Nonsevere CHD (n = 5713)	CHD vs no CHD	*P* value^a^	Severe vs Nonsevere CHD	*P* value^a^	Nonsevere CHD vs no CHD	*P* value^a^
**Health service use, mean (SD), No.**										
Outpatient obstetric visits^b^	7.1 (6.5)	8.2 (6.9)	8.0 (7.1)	8.2 (6.8)	0.16	<.001	−0.03	.56	0.17	<.001
Outpatient cardiologist visits	0.1 (0.6)	2.0 (2.8)	2.7 (3.1)	1.8 (2.6)	1.46	<.001	0.33	<.001	1.48	<.001
Outpatient other physician visits	6.3 (7.6)	8.8 (9.2)	8.5 (9.3)	8.9 (9.2)	0.33	<.001	−0.05	.34	0.34	<.001
Outpatient nonphysician encounters	4.7 (5.6)	6.0 (7.3)	5.3 (6.2)	6.2 (7.5)	0.21	<.001	−0.12	.008	0.24	<.001
Pharmacy claims	6.7 (7.6)	8.7 (9.5)	8.5 (9.0)	8.8 (9.6)	0.25	<.001	−0.03	.56	0.26	<.001
ED visits	0.5 (1.1)	0.8 (1.5)	0.8 (1.7)	0.8 (1.4)	0.24	<.001	0.02	.73	0.24	<.001
Inpatient visits	0.8 (0.6)	0.9 (0.7)	0.9 (0.7)	0.9 (0.7)	0.09	.001	0.02	.70	0.08	.006
Inpatient LOS^c^	2.8 (3.0)	4.0 (7.3)	4.7 (7.9)	3.9 (7.1)	0.23	<.001	0.09	.07	0.20	<.001
**Health care costs, median (IQR), US$**										
Total costs by setting^d^										
Outpatient physician	2311.9 (1266.2-4030.2)	4284.7 (2494.9-7010.7)	4769.7 (2681.0-7797.9)	4126.5 (2444.7-6815.7)	0.55	<.001	0.12	.003	0.53	<.001
Outpatient nonphysician^e^	1781.1 (663.4-4171.0)	4094.5 (1589.3-8940.5)	5224.1 (2125.7-11 436.6)	3839.7 (1504.3-8295.2)	0.42	<.001	0.22	<.001	0.37	<.001
Pharmacy	272.9 (85.7-795.0)	399.1 (131.8-1075.6)	320.8 (125.3-1000.7)	421.0 (133.2-1128.1)	0.14	<.001	0.02	.10	0.14	<.001
ED	1987.3 (1013.9-3806.3)	2443.4 (1278.3-4579.3)	2361.2 (1176.4-4405.4)	2460.5 (1282.3-4599.2)	0.18	<.001	−0.07	.74	0.20	<.001
Inpatient	14 337.7 (11 115.8-18 987.0)	16 256.4 (12 500.6-23 123.0)	17 436.7 (13 340.8-26 817.2)	15 984.7 (12 321.9-22 571.2)	0.32	<.001	0.15	.001	0.29	<.001
Total	18 469.0 (12 296.2-26 598.4)	25 843.8 (16 154.2-38 807.1)	29 350.9 (16 792.1-45 376.4)	25 174.9 (15 895.0-37 277.1)	0.46	<.001	0.17	<.001	0.43	<.001
OOP costs by setting^d^										
Outpatient physician	370.8 (127.4-827.6)	573.8 (248.5-1193.8)	562.1 (223.2-1166.7)	578.8 (253.6-1205.4)	0.32	<.001	−0.05	.28	0.34	<.001
Outpatient nonphysician^e^	235.8 (35.5-757.9)	418.2 (77.4-1167.5)	427.2 (92.8-1263.0)	412.9 (76.4-1140.6)	0.16	<.001	0.08	.24	0.15	<.001
Pharmacy	87.1 (28.8-228.6)	120.8 (40.1-283.8)	113.6 (37.6-253.2)	124.0 (40.5-290.9)	0.13	<.001	−0.05	.23	0.15	<.001
ED	242.8 (98.4-637.5)	238.7 (99.1-666.2)	222.5 (126.3-546.4)	245.2 (94.9-688.8)	0.003	.86	−0.04	.92	0.01	.91
Inpatient	1506.0 (367.9-2643.8)	1192.2 (184.0-2246.7)	1005.1 (116.0-2132.6)	1225.3 (200.5-2293.4)	−0.17	<.001	−0.09	.04	−0.15	<.001
Total OOP	2450.6 (851.1-4187.7)	2769.8 (1053.9-4668.8)	2610.6 (991.0-4505.1)	2794.3 (1068.2-4758.1)	0.14	<.001	−0.05	.27	0.15	<.001

Abbreviations: CHD, congenital heart disease; ED, emergency department; LOS, length of stay; OOP, out-of-pocket.

^a^ *P* values were obtained using the 2-tailed, unpaired *t* test or Wilcoxon rank sum test as appropriate.

^b^ Includes obstetric physician, birth center, and midwife visits.

^c^ The summary statistic is based on the patients who have any admissions in the pregnancy time range.

^d^ Component costs are calculated only for patients who had the service.

^e^ Total nonphysician outpatient costs and OOP costs include any outpatient non-ED and nonphysician visit but that occurred in the pregnancy time range.

**Figure 1.**
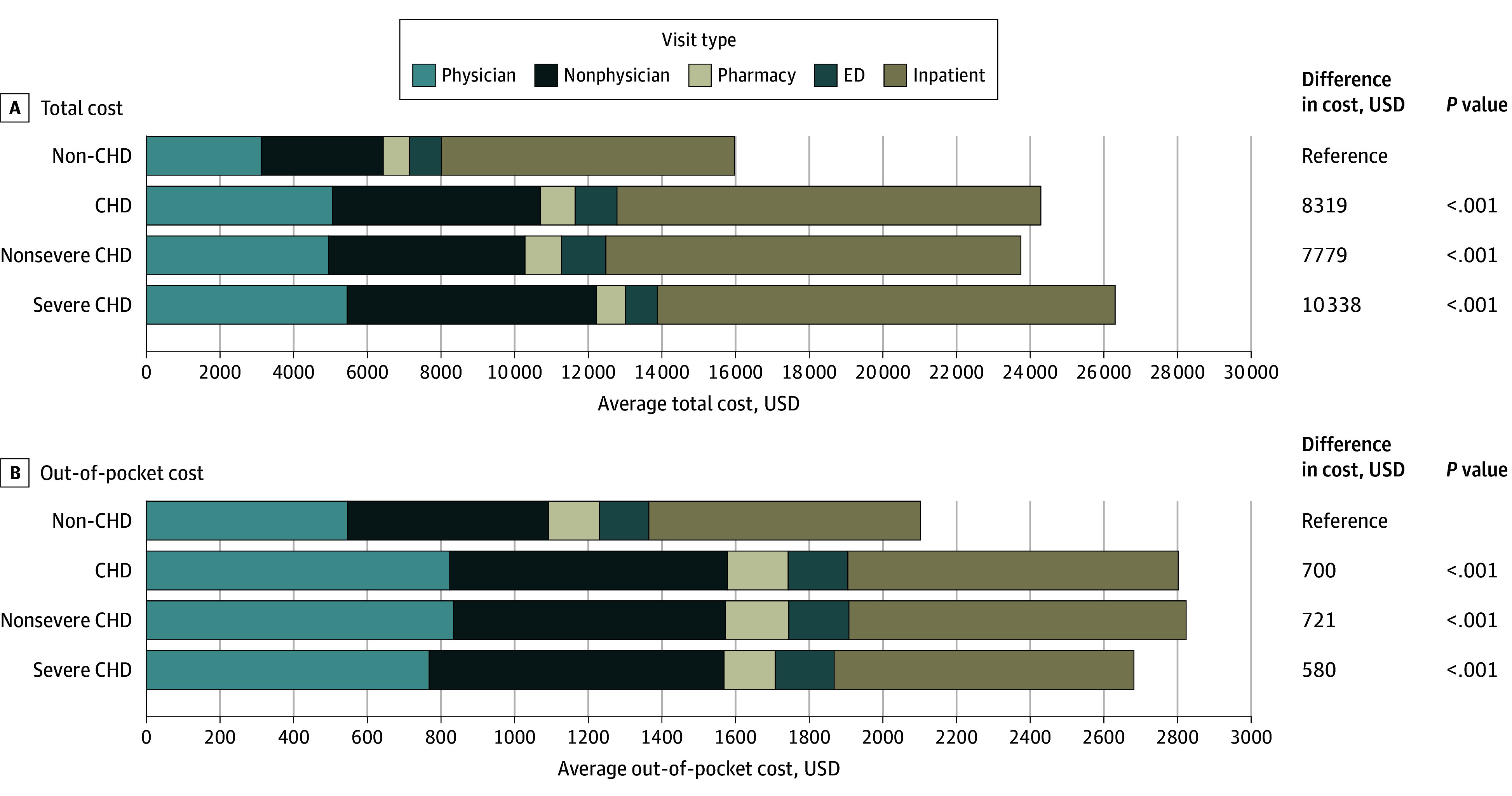
Adjusted Total and Out-of-Pocket Cost Differences During Pregnancy in Patients With and Without Congenital Heart Disease (CHD) Costs are adjusted for all baseline characteristics (age, US region, year of delivery, and insurance type), pregnancy-related events (obstetric conditions, cardiac conditions, noncardiac conditions, birth outcomes, and cesarean delivery), and significant interactions. For the total cost model, interactions were observed between live birth and 4 other variables: presence of CHD, region, cardiac conditions, and noncardiac conditions. For the OOP cost model, interactions were observed between age and 2 other variables: presence of CHD and region as well as the second order of age. ED indicates emergency department.

After adjustment for the covariates, having CHD was independently associated with higher total (adjusted cost ratio, 1.70; 95% CI, 1.57-1.84) and OOP (adjusted cost ratio, 1.40; 95% CI, 1.22-1.58) costs. The adjusted mean total costs per pregnancy were $15 971 (95% CI, $15 480-$16 461) for patients without CHD, $24 290 (95% CI, $22 773-$25 806) for patients with any CHD, $26 308 (95% CI, $22 788-$29 828) for patients with severe CHD, and $23 750 (95% CI, $22 110-$25 390) for patients with nonsevere CHD. This difference was significant between patients with and without CHD (difference, $8319; *P* < .001) (Figure 1A) but not between patients with severe vs nonsevere CHD. Similarly, the adjusted mean total OOP costs per pregnancy were $2102 (95% CI, $2036- $2168) for patients with no CHD vs $2802 (95% CI, $2621-$2984) for patients with any CHD (difference, $700; *P* < .001), $2682 (95% CI, $2296-$3068) for patients with severe CHD (difference, $580; *P* < .001), and $2824 (95% CI, $2622-$3026) for patients with nonsevere CHD (difference, $721; *P* < .001) (Figure 1B).

### Factors Associated With Health Care Use and Cost Among Patients With CHD

**Figure 2.**
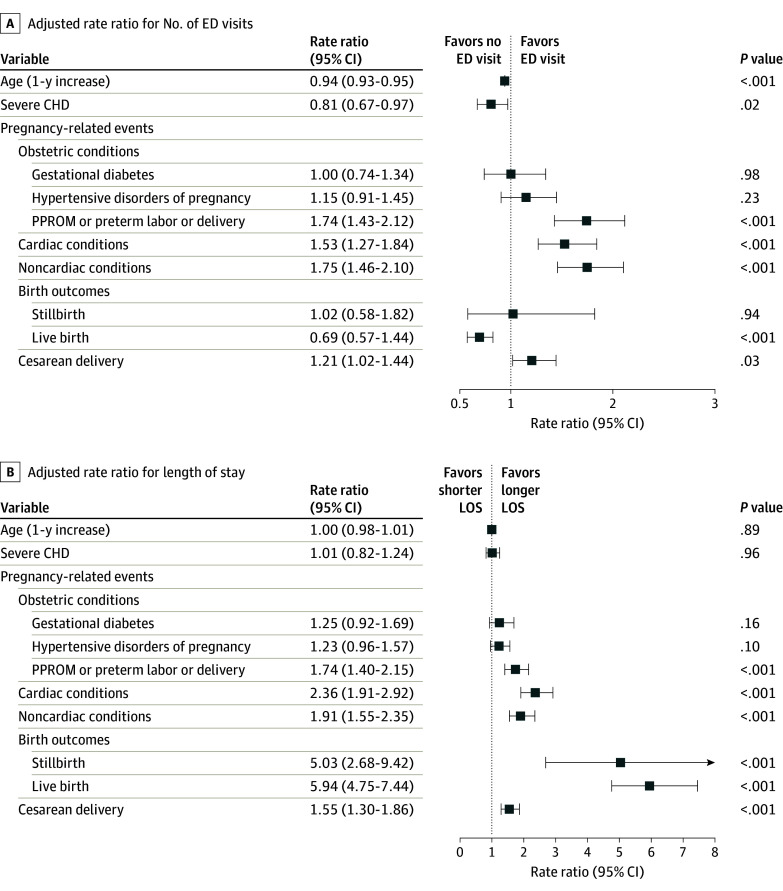
Variables Associated With Emergency Department (ED) Visits and Length of Stay During Pregnancy in Patients With Congenital Heart Disease (CHD) Variables are adjusted for all baseline characteristics (age, US region, year of delivery, and insurance type) and pregnancy-related events (obstetric conditions, cardiac conditions, noncardiac conditions, birth outcomes, and cesarean delivery). PPROM indicates preterm premature rupture of membrane.

**Figure 3.**
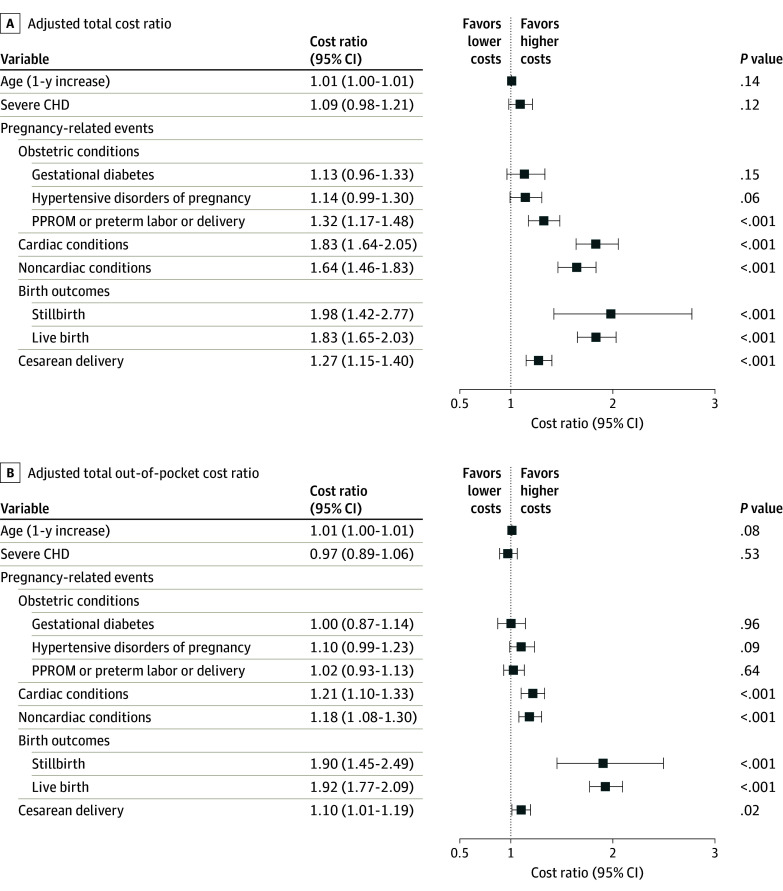
Variables Associated With Total and Out-of-Pocket Costs During Pregnancy in Patients With Congenital Heart Disease (CHD) Variables are adjusted for all baseline characteristics (age, US region, year of delivery, and insurance type) and pregnancy-related events (obstetric conditions, cardiac conditions, noncardiac conditions, birth outcomes, and cesarean delivery). PPROM indicates preterm premature rupture of membranes.

Among pregnancy-related events, PPROM, cardiac conditions, noncardiac conditions, and cesarean delivery were associated with higher ED visits; increasing age, severe CHD, and live birth were associated with lower ED visits, whereas there was no significant association of ED visits with gestational diabetes, hypertensive disorders of pregnancy, and stillbirth (Figure 2A). Only certain pregnancy-related events (specifically, PPROM, cardiac conditions, noncardiac conditions, stillbirth, live birth, and cesarean delivery) were independently associated with longer LOS during admission (Figure 2B). During the study period, total and OOP costs of pregnancy for patients with CHD were $58.0 million and $5.6 million, respectively (2024 adjusted costs are $76.1 million and $7.3 million, respectively). Except for obstetric conditions, all other pregnancy-related events were independently associated with higher total and OOP costs (Figure 3).

### Subanalysis

Overall findings were similar for pregnancies that resulted in live births (eAppendix 4, eTable 4, and eFigure 2 in Supplement 1). The adjusted mean total costs per live-birth pregnancy were $24 881 (95% CI, $24 536-$25 226) for patients without CHD, $31 399 (95% CI, $30 439-$32 359) for patients with any CHD, $33 733 (95% CI, $31 467-$36 000) for patients with severe CHD, and $30 834 (95% CI, $29 803-$31 866) for patients with nonsevere CHD. The adjusted mean OOP costs were $3020 (95% CI, $2955-$3086) for patients without CHD, $3551 (95% CI, $3370-$3732) for patients with any CHD, $3498 (95% CI, $3090-$3905) for patients with severe CHD, and $3564 (95% CI, $3365-$3762) for patients with nonsevere CHD.

## Discussion

This is the first study, to our knowledge, to provide pregnancy-related CHD anatomic severity–specific estimates of health care use and costs. We provide data that could allow better planning of future resource allocation for patients with CHD. First, patients with CHD during pregnancy, not surprisingly but likely appropriately, have significantly higher health care use, especially with outpatient cardiologists, than patients without CHD. Second, after adjustment for covariates, for each pregnancy, patients with CHD experience $8319 and $700 higher total and OOP costs, respectively, than those without CHD. Third, commercially insured patients with CHD incurred a substantial total and OOP cost of $58.0 million and $5.6 million, respectively, from January 1, 2010, to December 31, 2016 (2024 adjusted costs being $76.1 million and $7.3 million, respectively). Fourth, in patients with CHD, pregnancy-related events and not the anatomic severity of CHD were independently associated with higher total and OOP cost.

These results have several practice and policy implications for patients, clinicians, and policymakers involved in cardio-obstetric–related care. For patients with CHD, the OOP cost data could help in their personal financial planning. For example, knowledge about their expected OOP costs might help commercially insured adults with CHD in selecting a health insurance plan that will minimize their financial risk, especially during their childbearing years. Depending on the nature of insurance coverage, adults with CHD may be advised to take advantage of employee-provided health savings plans. It is important to counsel and guide patients with CHD in carefully reviewing and comparing the specific details of different insurance plans for their needs so they can personalize their choices of plan networks, coverage options, cost structures, and limitations. For clinicians, the study data could help in planning their clinic and training structure. Almost 40 000 to 50 000 patients with CHD enter adulthood every year,^1,25^ thus rapidly increasing the number of those reaching childbearing age, but these patients experience worse pregnancy outcomes than those without CHD.^3^ Because pregnancy-related events were independently associated with higher costs in our study, cardio-obstetricians can use this information to advocate for the required resources and workforce, eventually improving efficiency and quality of CHD care. For policymakers and payers, these data underscore the importance of considering the high health care needs and costs of the population of adults with CHD during pregnancy and use this information to design plans that are both affordable and appropriate. These learnings also could be extrapolated to adults with other childhood-onset lifelong illnesses and not only to those with CHD.

Our rates of pregnancy-related events are overall similar to those reported by Downing et al^3^ from a similar commercially insured database. Slight differences noted in the event rates are likely because of differences in data years (2010-2016 vs 2007-2014) and birth outcomes (abortions at <20 weeks’ gestation vs not) included. Although patients with severe CHD usually have worse outcomes than those with nonsevere CHD, both during and outside pregnancy, similar to the findings of Downing et al,^3^ we did not observe this difference in our study.^3,4,26,27^ Most obstetric and other medical conditions evaluated in this study usually occur later in the pregnancy, the period that patients with physiologically severe CHD are less likely to achieve because they either are counseled against pregnancy or undergo abortions before 20 weeks (whether induced or spontaneous). These findings also suggest that patients with anatomically severe CHD who have pregnancies that continue beyond 20 weeks likely represent a lower-risk subpopulation, with a pregnancy risk profile similar to that of patients with nonsevere CHD.

As cardio-obstetric physicians continue to manage and advocate for resources for the increasing population of patients with CHD through their pregnancies, our data highlight that it is important to consider the risk of pregnancy-related events in these patients, given their independent association with the service use and costs. Although we did not find anatomic CHD severity to be independently associated with service use and costs, future studies evaluating these outcomes by the recently described anatomic-physiologic classification of CHD^28^ might help to potentially identify CHD severity–specific high-risk patients. We observed, however, that increasing maternal age and higher anatomic severity of CHD were associated with significantly lower ED visit rates. We assume that this is because patients with increasing age and higher CHD severity are more likely to be followed up routinely by their physicians, resulting in better adherence and access to outpatient care, and are thus less likely to rely on ED care.^29,30^ This assumption was further demonstrated in our study by higher outpatient care costs but lower ED costs incurred by patients with severe vs nonsevere CHD.

To our knowledge, no prior data have described the cost estimates of pregnancy in patients with CHD in a community setting. Any prior national estimates for health care economic burden for patients with CHD have been mostly limited to inpatient data and have used patient bills (charges) as proxies for costs and do not provide information during pregnancy. Furthermore, charges used as a proxy for economic burden in prior studies may bear little resemblance to total economic cost and may lead researchers to draw unwarranted conclusions about actual resource consumption.^31,32^ Our estimates of the costs to the payers and patients in varied health care settings and data on factors associated with these costs are important for counseling, designing, and advocating for the resources needed for this increasing population of patients with CHD reaching childbearing age.

### Limitations

This study has some limitations inherent to the use of claims data. First, *ICD* codes have imperfect sensitivity and specificity; therefore, both CHD and pregnancy timing and outcomes might be misclassified. To minimize this possibility, we used previously validated codes and algorithms. For the CHD cohort, we erred on the side of being more specific than sensitive by limiting the age of the cohort to younger than 45 years and removing the codes known to be nonspecific.^14,15^ Overall, because the accuracy of the *ICD* codes is higher for severe anatomic CHD categories, our estimates are more relevant for the severe CHD subgroup. In addition, *ICD* codes only allow classifying CHD anatomically and not physiologically, thus limiting our ability to obtain functional severity-specific estimates. As with any other claims dataset, there is a time lag for data availability and analysis, resulting in our estimates being obtained from prior years, but we adjusted for inflation to provide 2024 estimates. Furthermore, irrespective of the years of data, our relative estimates provide important information about the additional resources that patients with CHD need compared with those without CHD. Finally, to enhance generalizability, our estimates from a commercially insured population should be supplemented with data from a population that uses other types of insurance, including government programs, and uninsured patients.

## Conclusions

We provide novel, community-based estimates of health service use and costs during pregnancy among commercially insured patients with CHD. We observed pregnancy-related events, but not anatomic CHD severity, to be independently associated with higher costs. These data provide evidence for the cardio-obstetric and CHD teams to plan for and advocate for effective care coordination models to improve care of the growing population of patients with CHD. Patients and payers can use these estimates to decide appropriate health care plans that can address the needs of these patients during childbearing age.

## Notes

Supplement 1. **eAppendix 1.** Congenital Heart Disease (CHD) Algorithm **eTable 1.** Congenital Heart Disease (CHD) Lesions and Their International Classification of Disease (ICD) Diagnostic Codes **eAppendix 2.** Pregnancy Algorithm **eTable 2.** Diagnostic and Procedure Codes Used for Birth Outcomes, Pregnancy Timings, and Cesarean Section **eAppendix 3.** Identifying Conditions: Obstetric, Cardiac, and Noncardiac **eTable 3.** Obstetric, Cardiac, and Noncardiac Conditions **eFigure 1.** Study Population **eAppendix 4.** Subanalysis of Patients With Livebirth Pregnancies **eTable 4.** Health Service Use and Costs Among Livebirth Pregnancies **eFigure 2.** Adjusted Total (2A) and Out-of-Pocket (2B) Cost Differences During Pregnancy With Livebirth in Patients With and Without Congenital Heart Disease
eReferences

Supplement 2.
Data Sharing Statement
