# Supplementary material for: Health Service Use and Costs During Pregnancy Among Privately Insured Individuals With Congenital Heart Disease
Source: JAMA Netw Open. 2024 May 13;7(5):e2410763. doi: 10.1001/jamanetworkopen.2024.10763 (PMC11091763; doi:10.1001/jamanetworkopen.2024.10763)
Supplement: Supplement 1. — eAppendix 1. Congenital Heart Disease (CHD) Algorithm eTable 1. Congenital Heart Disease (CHD) Lesions and Their International Classification of Disease (ICD) Diagnostic Codes eAppendix 2. Pregnancy Algorithm eTable 2. Diagnostic and Procedure Codes Used for Birth Outcomes, Pregnancy Timings, and Cesarean Section eAppendix 3. Identifying Conditions: Obstetric, Cardiac, and Noncardiac eTable 3. Obstetric, Cardiac, and Noncardiac Conditions eFigure 1. Study Population eAppendix 4. Subanalysis of Patients With Livebirth Pregnancies eTable 4. Health Service Use and Costs Among Livebirth Pregnancies eFigure 2. Adjusted Total (2A) and Out-of-Pocket (2B) Cost Differences During Pregnancy With Livebirth in Patients With and Without Congenital Heart Disease eReferences [file jamanetwopen-e2410763-s001.pdf]

Supplementary Online Content

Agarwal A, Duan R, Sobhani NC, Sabanayagam A, Marcus GM, Gurvitz M. Health service use and costs during pregnancy among privately insured individuals with congenital heart disease. *JAMA Netw Open*. 2024;7(5):e2410763. doi:10.1001/jamanetworkopen.2024.10763

eAppendix 1. Congenital Heart Disease (CHD) Algorithm
eTable 1. Congenital Heart Disease (CHD) Lesions and Their International Classification of Disease (ICD) Diagnostic Codes
eAppendix 2. Pregnancy Algorithm
eTable 2. Diagnostic and Procedure Codes Used for Birth Outcomes, Pregnancy Timings and Cesarean Section
eAppendix 3. Identifying Conditions: Obstetric, Cardiac, and Noncardiac
eTable 3. Obstetric, Cardiac, and Noncardiac Conditions
eFigure 1. Study Population
eAppendix 4. Subanalysis of Patients With Livebirth Pregnancies
eTable 4. Health Service Use and Costs Among Livebirth Pregnancies
eFigure 2. Adjusted Total (2A) and Out-of-Pocket (2B) Cost Differences During Pregnancy With Livebirth in Patients With and Without Congenital Heart Disease
eReferences

This supplementary material has been provided by the authors to give readers additional information about their work.

© 2024 Agarwal A et al. *JAMA Network Open.*

eAppendix 1. Congenital Heart Disease (CHD) Algorithm

Patients were identified as having CHD if they had an International Classification of Disease, Ninth (ICD-9) and Tenth (ICD-10) Revision diagnosis code for any CHD lesion (**eTable 1**).[1–4] If an ICD code for CHD was present on ≥2 outpatient claims separated by >30 days or ≥1 inpatient claim at any billing position during the period of enrollment, these patients were then considered to have CHD. For patients with codes for more than one CHD diagnosis, we used the hierarchical algorithm proposed by Broberg et al.[4] to designate one condition per patient as their principal CHD diagnosis. The principle of the hierarchy is that physiologically more severe or complex lesions take precedence over less severe or simpler ones. We excluded any patients who had CHD diagnoses codes documented only during pregnancy or delivery-related claims (see below for the pregnancy algorithm) to avoid inclusion of pregnant women with fetuses affected by CHD. We also excluded claims with ICD codes for nonspecific CHD diagnosis such as atrial septal defect (due to lack of differentiation with patent foramen ovale); unspecified congenital heart, pulmonary artery, aortic, or great vein anomalies; and congenital heart block.[3,4] The remaining patients were categorized based on their anatomic severity as 1) Severe CHD: Eisenmenger Syndrome, single ventricle, hypoplastic left heart syndrome, transposition of great arteries, Tetralogy of Fallot, truncus arteriosus, and endocardial cushion defect; and 2) Nonsevere CHD: coarctation of aorta, anomalies of the pulmonary artery or pulmonary valve, anomalies of the tricuspid valve (including Ebstein's anomaly), ventricular septal defect, patent ductus arteriosus, unspecified septal defects, anomalous pulmonary venous return, subaortic stenosis, aortic anomalies, anomalies of the aortic or mitral valve, anomalous coronary artery and other congenital heart anomaly.

© 2024 Agarwal A et al. *JAMA Network Open.*

**eTable 1.** Congenital Heart Disease (CHD) Lesions and Their International Classification of Disease (ICD) Diagnostic Codes		
	**ICD 9 codes**	**ICD 10 codes**
**Severe CHD**		
Eisenmenger (CHD code AND cyanosis)	782.5 PLUS other congenital code (782.5+745-747)	I27.83
Hypoplastic left heart syndrome	746.7	Q23.4
Single ventricle	745.3, 746.1	Q20.4, Q22.4, Q22.6,
Transposition Complex	745.1, 745.10, 745.11, 745.12, 745.19	Q20.1, Q20.2, Q20.3, Q20.5, Q20.8
Tetralogy of Fallot	745.2, 745.4 AND 746.00, 746.01, or 746.02 or 746.09	Q21.3, Q21.0 AND Q22.0 or Q22.1 or Q22.2 or Q22.3
Truncus Arteriosus	745.0	Q20.0
Endocardial Cushion Defect	745.60, 745.61,745.69	Q21.2
**Nonsevere CHD**		
Coarctation of aorta/ Interrupted aortic arch	747.1, 747.10, 747.11	Q25.1, Q25.21
Anomalies of the pulmonary artery (except pulmonary atresia)/ pulmonary valve	746.00, 746.02, 746.09, 747.31, 747.39	Q25.79, Q25.5, Q25.71, Q22.1, Q22.2, Q22.3
Ebstein's anomaly/ anomalies of tricuspid valve	746.2	Q22.5, Q22.8, Q22.9
Shunts: Ventricular septal defect, patent ductus arteriosus, unspecified defect of septal closure, aortopulmonary window, Anomalous pulmonary venous return	745.4, 747.0, 747.41, 747.42, 745.9	Q21.0, Q21.4, Q21.9, Q25.0, Q26.2, Q26.3
Subaortic Stenosis	746.81	Q24.4
Aortic Anomalies	747.22	Q25.29, Q25.41, Q25.42, Q25.43, Q25.44, Q25.48
Anomalies of the aortic or mitral valve	746.3, 746.4, 746.5, 746.6	Q23.0, Q23.1, Q23.2, Q23.3, Q23.8, Q33.9
Anomalous coronary artery	746.85	Q24.5
Other congenital heart anomaly	745.7, 746.82, 746.83, 747.9	Q20.9, Q24.2, Q24.3, Q24.6, Q28.9
Atrial septal defect (excluded from the analytic sample)	745.5, 745.8	Q21.1, Q21.8

© 2024 Agarwal A et al. *JAMA Network Open.*

eAppendix 2. Pregnancy Algorithm

We applied the published algorithm by Ailes et al[5] that uses pregnancy-related procedure and diagnostic codes indicating end of a pregnancy to assign birth outcome, estimate gestational age (GA) at end of pregnancy, and used estimated GA to calculate date of last menstrual period (LMP, **eTable2**). Since Ailes et al primarily used ICD 9 codes, we used the corresponding ICD 10 codes from other validated studies.[6,7] Briefly, we used inpatient and outpatient services files to identify all the claims that mapped to one of the diagnosis, procedure, or Diagnosis Related Group (DRG) codes indicative of a birth outcome (**eTable 2**). We prioritized the codes in the following order: 1) diagnosis codes, 2) DRG codes, and 3) procedure codes. If birth outcomes were still discrepant, we assigned one using the following hierarchy: 1) stillbirth, 2) live birth, 3) induced abortion, 4) spontaneous abortion, 5) abortion of unknown type, and 6) ectopic pregnancy. We assigned GA using the records with the selected birth outcome. If more than one GA estimate was present, we used the minimum GA to avoid overestimating exposure during pregnancy. If a visit indicated a full-term delivery without a code indicating a more specific GA, we estimated that to occur at 40 weeks of gestation. LMP was estimated as the admission (for inpatient visits) or service (for outpatient visits) date minus the GA. To differentiate visits associated with separate pregnancies, we required ≥ 2 months between one birth outcome and the LMP of the next pregnancy. For those pregnancies that could not be differentiated, we prioritized the inpatient followed by outpatient service claims and applied the above hierarchy of diagnosis/ procedure codes, then re-assigned birth outcomes, determined GA as above and estimated the LMP. Multiple births were included, but, due to the limitations of claims data, we could not distinguish a multiple birth from a singleton birth unless the birth outcomes differed between the multiples (e.g, combination of live birth[s] and stillbirth[s]).

To capture the entire pregnancy time range, we only included pregnancies that were completely enrolled in a contributing commercial insurance from 60 days before LMP through 90 days after delivery. Pregnancies conceived in 2009 and ending in 2010 or conceived in 2016 and ending in 2017 were thus excluded in the analytic sample. To differentiate visits associated with separate pregnancies,

© 2024 Agarwal A et al. *JAMA Network Open.*

we required ≥ 2 months between one birth outcome and the LMP of the next pregnancy. In case of any overlap between two pregnancies, the associated visits were included in the previous pregnancy.

© 2024 Agarwal A et al. *JAMA Network Open.*

**eTable 2.** Diagnostic and Procedure Codes Used for Birth Outcomes, Pregnancy Timings and Cesarean Section						
**Birth Outcomes**	**GA[a]**	**ICD 9**	**ICD 10**	**CPT**	**HCPC**	**DRG**
Abortion (induced or spontaneous)	8-33 weeks	Diagnostic codes: 631.xx, 632.xx, 634.xx-637.xx Procedure codes: 69.01, 69.51, 69.52, 74.91, 75.0	Diagnostic codes: O01, O03, O04, O020, O021, O0289, O029, Z332 Procedure codes: 10A00ZZ, 10A03ZZ, 10A04ZZ, 10A07ZX, 10A07ZZ, 10A08ZZ, 10D17ZZ, 10D18ZZ	59820, 59830, 59840, 59841, 59850-59852, 59855-59857, 59812, 59830, 59821	S0199, S2260, S2265, S2262, S2266, S2267	770, 779
Ectopic pregnancy	8 weeks	Diagnostic codes: 633.xx Procedure codes: 66.62, 74.3	Diagnostic codes: O00, O08, O008, Procedure codes: 10T20ZZ, 10T23ZZ, 10T24ZZ, 10T27ZZ, 10T28ZZ	59100, 59120, 59121, 59130, 59135, 59136, 59140, 59150		777
Livebirth	23-43 weeks	Diagnostic codes: 644.21, 645.11, 645.21, 649.81, 649.82, 650, 651.01, 651.11, 651.21, 669.70, 669.71, 765.0X[c], 765.10[c]-765.29[c], 766.21[c], 766.22[c], V270, V27.2, V27.5, V30.0[c], V30.00[c], V30.01[c], V31.0[c], V31.00[c], V31.01[c], V33[c], V33.0[c], V33.00[c], V33.01[c], V34.0[c], V34.00[c], V34.01[c], V37[c], V37.0[c], V37.00[c], V37.01[c], V39.0[c], V39.00[c], V39.01[c] Procedure Codes: 72.0, 72.1, 72.2, 72.21, 72.29, 72.3, 72.31, 72.39, 72.4,	Z370, Z372, Z3750-Z3754, Z3759, O6012X0, O6012X1, O6012X2, O6012X3, O6012X4, O6012X5, O6012X9, O6013X0, O6013X1, O6013X2, O6013X3, O6013X4, O6013X5, O6013X9, O6014X0, O6014X1, O6014X2, O6014X3, O6014X4, O6014X5, O6014X9, O6022X0, O6022X1, O6022X2, O6022X3, O6022X4, O6022X5, O6022X9, O6023X0, O6023X1, O6023X2, O6023X3, O6023X4, O6023X5, O6023X9, O7582, O80, O82 Procedure Codes: 0W8NXZZ, 10A07Z6, 10D00Z0, 10D00Z1, 10D00Z2, 10D07Z3,	59612, 59614, 59620		790[c]-793[c], 795[c], 765-768, 774, 775

© 2024 Agarwal A et al. *JAMA Network Open.*

		72.5, 72.51-72.54, 72.6, 72.7, 72.71, 72.79, 72.8, 72.9, 73.0, 73.01, 73.09, 73.1, 73.2, 73.22, 73.3, 73.4, 73.5, 73.51, 73.59, 73.6, 73.8, 73.9, 73.91-73.94, 73.99, 74, 74.0-74.2, 74.4, 74.9, 74.99	10D07Z4, 10D07Z5, 10D07Z6, 10D07Z7, 10D07Z8, 10E0XZZ			
Stillbirth	28-36 weeks	Diagnostic codes: 656.4x, V27.1, V27.4, V27.7	Z371, Z374, Z377, O364XX0- O364XX5, O364XX9			
Livebirth and Stillbirth	31-36 weeks	Diagnostic codes: 651.31, 651.41, 651.51, V27.3, V27.6, V32.0[c], V32.00[c], V32.01[c], V35.0[c], V35.00[c], V35.01[c], V36.0[c], V36.00[c], V36.01[c]	Z373, Z3760-Z3764, Z3769			
Caesarean section		Diagnostic codes: 649.81, 649.82, 669.70, 669.71, V30.01[c], V31.01[c], V32.01[c], V3301[c], V3401[c], V35.01[c], V37.01[c], V39.01[c] Procedure Codes: 74, 74.0, 74.1, 74.2, 74.4, 74.9, 74.99	Diagnostic codes: O7582, O82 Procedure Codes: 10D00Z0, 10D00Z1, 10D00Z2	59620		765, 766

[a] If a claim or visit included codes with different pregnancy outcomes, we prioritized code types in the following order: 1) information from diagnosis codes, 2) information from DRG codes, and 3) information from procedure codes. If pregnancy outcomes were still discrepant, we assigned a pregnancy outcome using the following hierarchy: 1) stillbirth, 2) live birth, 3) induced abortion, 4) spontaneous abortion, 5) abortion of unknown type, and 6) ectopic pregnancy.
[b] Unless the visit had a code indicating a pre- or post-term delivery
[c] Infant code
Abbreviations: GA=gestational age, ICD = International Classification of Diseases, CPT = Current Procedural Terminology, HCPC=Healthcare Common Procedure Coding System codes, DRG= Diagnosis Related Groups

© 2024 Agarwal A et al. *JAMA Network Open.*

eAppendix 3. Identifying Conditions: Obstetric, Cardiac, and Noncardiac

We used the ICD codes and algorithms described previously to identify obstetric conditions (gestational diabetes mellitus [GDM], hypertensive disorders of pregnancy, or preterm premature rupture of membranes [PPROM] or preterm labor/delivery)[7–10] for patients whose pregnancy lasted for more than 20 weeks of gestation. Women with a diabetes mellitus diagnosis code (ICD 9: 250, 648.0, 790.2; ICD10: O24.0, O24.1, O24.3, O24.8, E08-E13, R73.0, or R73.9) from 2010 to 2016 before a GDM diagnosis code were not classified as having GDM; as previously described.[9]

Several tools are available to identify medical conditions in administrative data.[11] We used the ICD codes as listed by the Agency for Healthcare Research and Quality (AHRQ) Elixhauser comorbidity measures to identify majority of the cardiac and noncardiac conditions during pregnancy (**eTable 3**).[12] The AHRQ measure however does not include some conditions that are important in CHD and/or pregnancy, so we used the AHRQ's single-level Clinical Classification System (CCS)[13] codes to identify the ICD codes for determining conditions such as coronary artery disease, stroke, deep venous thrombosis, pulmonary embolism, and infective endocarditis. The CCS provides a way to classify diagnoses and procedures into a limited number of categories by aggregating individual ICD codes into broad diagnosis and procedure groups to facilitate statistical analysis and reporting. For determining coronary dissection, peripartum cardiomyopathy and seizure during pregnancy, we used the previously published ICD 9 codes[8] and supplemented the corresponding ICD 10 codes using the CCS classification. All the conditions, except obstetric conditions, were identified if documented during the pregnancy time range i.e. from 60 days before the last menstrual period to 90 days postpartum per pregnancy; we did not assess whether any of the condition was first diagnosed during pregnancy. Similar to another study, for deep vein thrombosis and pulmonary embolism, we required ≥1 inpatient diagnosis code or ≥1 anticoagulation outpatient prescription claim documented within 90 days of an outpatient diagnosis code.[14] All other conditions were identified by ≥1 inpatient diagnosis code or ≥2 outpatient diagnosis codes separated by ≥1 day.

© 2024 Agarwal A et al. *JAMA Network Open.*

**eTable 3.** Obstetric, Cardiac, and Noncardiac Conditions		
**Conditions**	**ICD 9 codes**	**ICD 10 codes**
**Obstetric ≥ 20 weeks of gestation**		
Gestational diabetes mellitus	648.8x	O24.4x
Hypertensive disorders of pregnancy (Preeclampsia, eclampsia, or gestational hypertension)	642.3X-642.7X	O11.1-O11.5, O11.9, O13.1-O13.5, O13.9, O14.0, O14.00, O14.02-O14.05, O14.1, O14.10, O14.12-O14.15, O14.2, O14.20, O14.22-O14.25, O14.9, O14.90, O14.92-O14.95, O15.00, O15.02, O15.03, O15.1, O15.2, O15.9, O16.4
Preterm labor or preterm delivery	644.x	O6010X0–9, O6012X0–9, O6013X0–9, O6014X0–9, O6020X0–9, O6022X0–9, O6023X0–9
Preterm premature rupture of membranes	658.1x	O42011–9, O42111–9, O42911–9, O4202, O4292
**Cardiac Conditions**		
Congestive heart failure	398.91, 402.01, 402.11, 402.91, 404.01, 404.03, 404.11, 404.13, 404.91, 404.93, 425.4-425.9, 428.x	I09.9, I11.0, I13.0, I13.2, I25.5, I42.0, I42.5-I42.9, I43.x, I50.x, P29.0
Cardiac arrhythmias	426.0, 426.13, 426.7, 426.9, 426.10, 426.12, 427.0-427.4, 427.6-427.9, 785.0, 996.01, 996.04, V45.0, V53.3	I44.1-I44.3, I45.6, I45.9, I47.x-I49.x, ROO.O, ROO.1, ROO.8, T82.1, Z45.0, Z95.0
Acute myocardial infarction or chronic coronary artery disease	410.x, 411.x-414.x, V458.1, V458.2	I21, I22, I25
Coronary dissection	414.12	I25.42
Peripartum cardiomyopathy	425.x, 674.5x	O90.3
**Noncardiac conditions**		
Stroke or cerebrovascular disorders	346.6x, 430.x-438.x	I60-I63, I65-I6, I69
Coagulopathy	286.x, 287.1, 287.3-287.5	D65x.-D68.x, D69.1, D69.3- D69.6
Anemia	280.0, 280.1-281.9, 281.x	D50.0, D50.8, D50.9, D51.x-D53.x
Liver disease	070.22, 070.23, 070.32, 070.33, 070.44, 070.54, 070.6, 070.9, 456.0-456.2, 570.x, 571.x, 572.2-572.8, 573.3, 573.4, 573.8, 573.9, V42.7	B18.x, I85.x, I86.4, I98.2, K70.x, K71.1, K71.3-K71.5, K71.7, K72.x-K74.x, K76.0, K76.2-K76.9. Z94.4
Chronic pulmonary disease	416.8, 416.9, 490.x-505.x, 506.4, 508.1, 508.8	I27.8, 127.9, J40.x-J47.x, J60.x-J67.x, J68.4, J70.1, J70.3
Deep venous thrombosis	451.x-453.x, 671.3X, 671.4X, 671.5X, 671.9X	I80-I82
Pulmonary embolism	415.1x	I26
Infective endocarditis	421.0, 421.1, 421.9, 424.90, 424.91, 424.99	I33.0, I33.9, I38
Seizure	345	G40

© 2024 Agarwal A et al. *JAMA Network Open.*

eFigure 1. Study Population
CHD= congenital heart disease

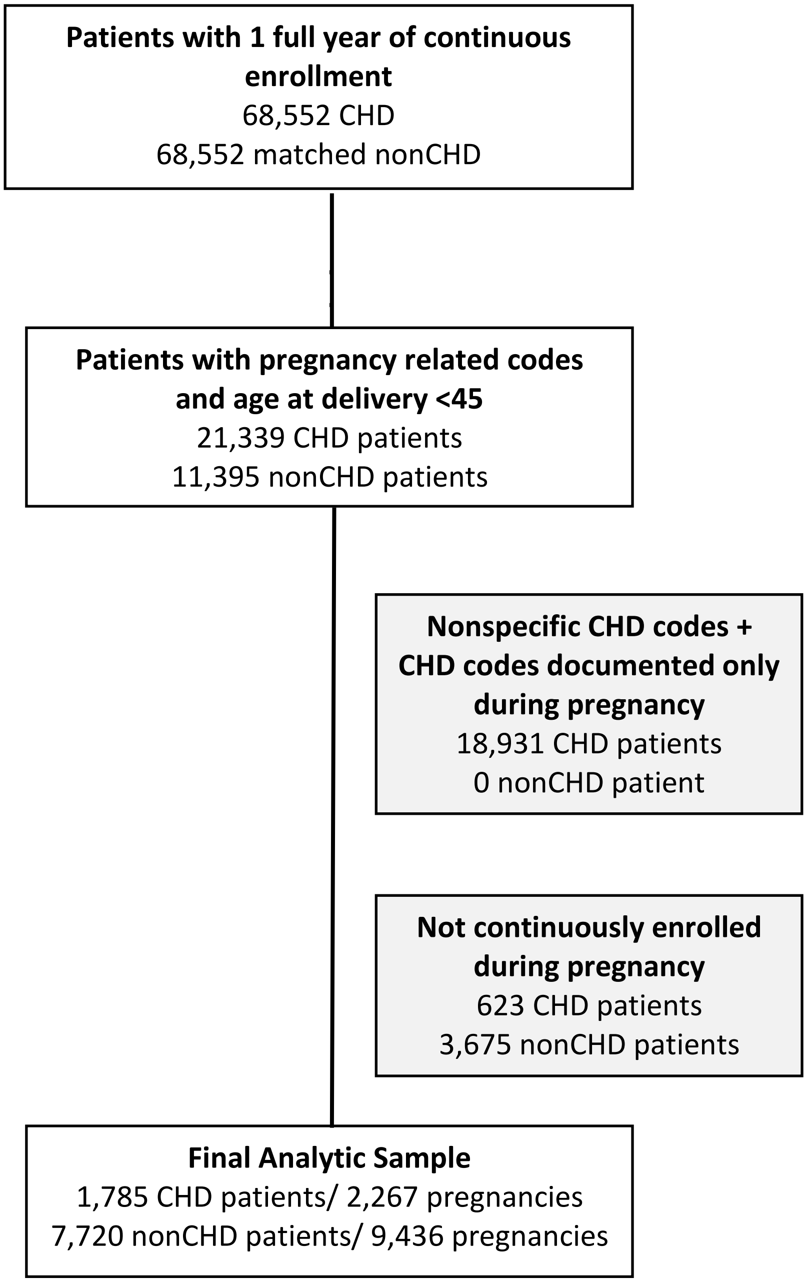

© 2024 Agarwal A et al. *JAMA Network Open.*

eAppendix 4. Subanalysis of Patients With Livebirth Pregnancies

Between 2010 and 2016, there were 1,418 patients with CHD contributing 1,649 pregnancies (348 pregnancies among 302 patients with severe CHD and 1,301 pregnancies among 1,116 patients with nonsevere CHD) and 6,419 patients without CHD contributing 7,275 pregnancies. The mean (standard deviation [SD]) patient age was 31.4 (5.1) years.

Compared to nonCHD, pregnancies in patients with CHD had significantly higher healthcare use in all categories (standardized mean differences [SMDs] ranging from 0.19 [p<0.001] for inpatient admissions to 1.72 [p<0.001] for outpatient cardiologist visits) and significantly higher costs in all categories (SMDs ranging from 0.15 [p<0.001] for ED visits to 0.63 [p<0.001] for outpatient physician visits) except for out-of-pocket inpatient and out-of-pocket emergency department costs (**eTable 4**). Compared to nonsevere CHD, pregnancies in patients with severe CHD had significantly higher outpatient cardiology visits, lower outpatient nonphysician encounters, and higher costs (except for similar pharmacy and emergency department costs) while out-of-pocket costs were not significantly different except for lower inpatient out-of-pocket costs (**eTable 4**). After adjusting for baseline and pregnancy-related event variables, having CHD was independently associated with higher total (adjusted cost ratio 1.26, 95% confidence interval [CI], 1.21-1.31) and out-of-pocket pregnancy costs (adjusted cost ratio of 1.17, 95% CI, 1.11-1.24). The adjusted mean total cost per pregnancy were $24,881 (95%CI, $24,536-$25,226) for patients without CHD, $31,399 (95%CI, $30,439-$32,359) for patients with any CHD, $33,733 (95%CI, $31,467-$36,000) for patients with severe CHD, and $30,834 (95%CI, $29,803-$31,866) for patients with nonsevere CHD respectively. This difference was significant between nonCHD and CHD patients (**eFigure 2A**) but not between severe and nonsevere CHD patients (p=0.20). Similarly, the adjusted mean total out-of-pocket cost per pregnancy were $3,020 (95%CI, $2,955- $3,086) for patients without CHD, $3,551 (95%CI, $3,370- $3,732) for patients with any CHD, $3,498 (95%CI, $3,090-$3,905) for patients with severe CHD, and $3,564 [95%CI, $3,365-$3,762) for patients with nonsevere CHD respectively (**eFigure 2B**).

© 2024 Agarwal A et al. *JAMA Network Open.*

eTable 4. Health Service Use and Costs Among Livebirth Pregnancies							
Variable	Pregnancies in patients without CHD (n=9,438)	Pregnancies in patients with CHD			Standardized Mean Difference (P value)[e]		
		Any CHD (n=6,385)	Severe CHD (n=672)	Nonsevere CHD (n=5,713)	CHD vs. no CHD	Severe vs. Nonsevere CHD	Nonsevere CHD vs. no CHD
**Health service use, mean (SD), No.**							
Outpatient obstetric visits[a]	8.2 (6.7)	9.8 (7.1)	9.8 (7.5)	9.8 (7.0)	0.24 (<.001)	-0.001 (0.98)	0.24 (<.001)
Outpatient cardiologist visits	0.1 (0.6)	2.4 (2.8)	3.2 (3.3)	2.1 (2.6)	1.72 (<.001)	0.40 (<.001)	1.76 (<.001)
Outpatient other physician visits	6.8 (8.1)	9.8 (9.9)	9.5 (10.1)	9.8 (9.9)	0.36 (<.001)	-0.04 (0.55)	0.37 (<.001)
Outpatient nonphysician encounters	5.2 (5.8)	6.8 (7.8)	6.0 (6.6)	7.0 (8.1)	0.25 (<.001)	-0.14 (0.01)	0.29 (<.001)
Pharmacy claims	7.4 (8.0)	9.8 (10.1)	9.5 (9.7)	9.9 (10.2)	0.29 (<.001)	-0.04 (0.56)	0.30 (<.001)
ED visits	0.5 (1.1)	0.8 (1.5)	0.9 (1.8)	0.8 (1.4)	0.22 (<.001)	0.06 (0.37)	0.21 (<.001)
Inpatient visits	1.0 (0.4)	1.1 (0.5)	1.2 (0.5)	1.1 (0.5)	0.19 (<.001)	0.08 (0.22)	0.17 (<.001)
Inpatient LOS[b]	2.8 (3.0)	3.9 (6.8)	4.7 (8.1)	3.7 (6.4)	0.27 (<.001)	0.14 (0.04)	0.24 (<.001)
**Health care costs, median (IQR), US$**							
Total costs by setting[c]							
Outpatient physician	2,532.5 (1398.0-4308.9)	4,888.3 (3035.5-7687.2)	5,356.2 (3,396.0-8,714.0)	4,736.4 (2,959.6-7,510.0)	0.63 (<.001)	0.12 (0.001)	0.61 (<.001)
Outpatient nonphysician[d]	1,834.8 (743.2-4062.4)	4,342.6 (1,919.8-9,195.1)	5,755.5 (2,384.0-12,152.0)	4,122.5 (1,798.6-8655.8)	0.44 (<.001)	0.20 (<.001)	0.40 (<.001)
Pharmacy	302.1 (96.3- 859.7)	447.5 (158.1-1,186.8)	376.6 (148.0-1,091.6)	476.8 (163.0-1,193.9)	0.16 (<.001)	0.08 (0.16)	0.14 (<.001)
ED	1,884.2 (954.7-3,599.0)	2,313.1 (1082.3-4,285.0)	2,379.3 (1,100.7-4,683.7)	2,289.0 (1,080.7-4,230.5)	0.15 (<.001)	0.03 (0.53)	0.15 (0.002)
Inpatient	14,364.3 (11,179.9-19,003.0)	16,261.3 (12,693.0-22,829.6)	17,511.9 (13,770.0-26,226.9)	15,965.6 (12,392.1-22,274.2)	0.27 (<.001)	0.20 (<.001)	0.23 (<.001)
Total	20,953.3 (15,871.0-29,089.6)	30,042.1 (21,937.2-43,024.9)	35,617.1 (23,495.8-50,905.8)	29,102.3 (21,636.4-40,726.3)	0.51 (<.001)	0.25 (<.001)	0.46 (<.001)
Out-of-pocket costs by setting[c]							
Outpatient Physician	411.1 (145.4-877.1)	637.9 (294.2-1,325.1)	634.0 (276.0-1,277.1)	638.4 (298.2-1,335.6)	0.38 (<.001)	-0.05 (0.47)	0.40 (<.001)

© 2024 Agarwal A et al. *JAMA Network Open.*

Outpatient nonphysician[d]	253.0 (41.2- 768.6)	479.6 (116.6- 1,215.5)	504.7 (118.1- 1,266.2)	475.5 (116.5- 1,195.1)	0.19 (<.001)	0.09 (0.54)	0.18 (<.001)
Pharmacy	96.5 (32.4- 248.4)	135.2 (47.1- 320.6)	117.0 (43.7- 255.0)	139.3 (47.7- 337.2)	0.15 (<.001)	-0.04 (0.15)	0.16 (<.001)
ED	221.0 (95.6- 592.0)	233.6 (95.8- 592.0)	228.4 (117.0- 577.5)	235.7 (94.4- 612.0)	0.04 (0.51)	0.04 (0.55)	0.03 (0.73)
Inpatient	1,510.9 (372.9- 2,647.4)	1,214.3 (191.0- 2,282.6)	986.5 (113.7- 2,151.6)	1,250.7 (235.6- 2,314.6)	-0.16 (<.001)	-0.09 (0.03)	-0.14 (<.001)
Total out-of-pocket	2,971.8 (1,397.9 4,595.5)	3,404.5 (1,682.1- 5,209.7)	3,329.8 (1,529.9- 4,950.3)	3423.0 (1,740.3- 5,244.8)	0.17 (<.001)	-0.04 (0.28)	0.18 (<.001)

Abbreviations: CHD = congenital heart defects, ED = emergency department, SD = standard deviation, IQR = interquartile range
[a]Includes Obstetric physician, birth center and midwife visits.
[b]The summary statistic is based on the patients who have any admissions in the pregnancy time range.
[c]Component costs are calculated only for patients who had the service.
[d]Total Nonphysician outpatient costs and out-of-pocket costs includes any outpatient visit which is non-ED and nonphysician but that occurred in the pregnancy time range.
[e]p values were obtained using student t-test or Wilcoxon rank sum test as appropriate.

© 2024 Agarwal A et al. *JAMA Network Open.*

eFigure 2. Adjusted Total (2A) and Out-of-Pocket (2B) Cost Differences During Pregnancy With Livebirth in Patients With and Without Congenital Heart Disease

Adjusted for all baseline characteristics (age, US region, year of delivery, insurance type), pregnancy-related events (obstetric conditions, cardiac conditions, noncardiac conditions, birth outcomes, caesarean section) and significant interactions if observed. For total cost model, no interactions were observed. For out-of-pocket cost model, interactions were observed between age and region.

Abbreviations: CHD=congenital heart disease; USD=Unites States dollars; ED=emergency department, OOP=out-of-pocket cost

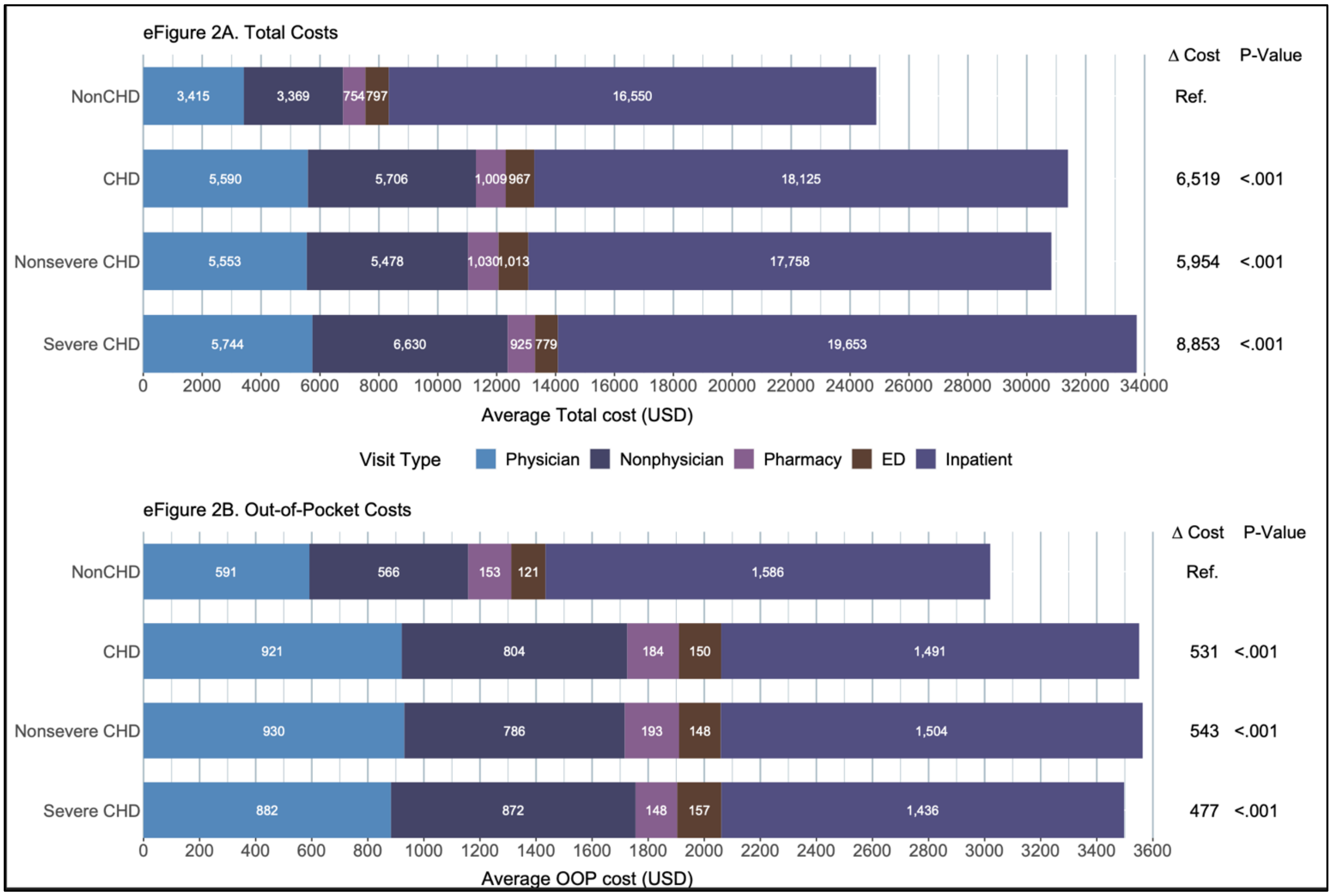

© 2024 Agarwal A et al. *JAMA Network Open.*

eReferences

1. Agarwal A, Thombley R, Broberg CS, et al. Age-and Lesion-Related Comorbidity Burden Among US Adults With Congenital Heart Disease: A Population-Based Study. *J Am Heart Assoc*. 2019;8(20):e013450.

2. Agarwal A MG Vittinghoff E, Myers JJ, Dudley RA, Khan A, John AS. Ambulatory Healthcare Service Utilization and Costs Among Commercially Insured U.S. Adults with Congenital Heart Disease. *Publ Online Sept 30 2020 JAMA Netw Open*. Published online 2020. doi:10.1001/jamanetworkopen.2020.18752

3. Ivey LC, Rodriguez FH, Shi H, et al. Positive Predictive Value of International Classification of Diseases, Ninth Revision, Clinical Modification, and International Classification of Diseases, Tenth Revision, Clinical Modification, Codes for Identification of Congenital Heart Defects. *J Am Heart Assoc*. 2023;12(16):e030821. doi:10.1161/JAHA.123.030821

4. Broberg C, McLarry J, Mitchell J, et al. Accuracy of administrative data for detection and categorization of adult congenital heart disease patients from an electronic medical record. *Pediatr Cardiol*. 2015;36(4):719-725.

5. Ailes EC, Simeone RM, Dawson AL, Petersen EE, Gilboa SM. Using insurance claims data to identify and estimate critical periods in pregnancy: an application to antidepressants. *Birt Defects Res A Clin Mol Teratol*. 2016;106(11):927-934.

6. Moll K, Wong HL, Fingar K, et al. Validating Claims-Based Algorithms Determining Pregnancy Outcomes and Gestational Age Using a Linked Claims-Electronic Medical Record Database. *Drug Saf*. 2021;44(11):1151-1164. doi:10.1007/s40264-021-01113-8

7. Chomistek AK, Phiri K, Doherty MC, et al. Development and Validation of ICD-10-CM-based Algorithms for Date of Last Menstrual Period, Pregnancy Outcomes, and Infant Outcomes. *Drug Saf*. 2023;46(2):209-222. doi:10.1007/s40264-022-01261-5

8. Downing KF, Tepper NK, Simeone RM, et al. Adverse pregnancy conditions among privately insured women with and without congenital heart defects. *Circ Cardiovasc Qual Outcomes*. 2020;13(6):e006311.

9. Hsu S, Selen DJ, James K, et al. Assessment of the Validity of Administrative Data for Gestational Diabetes Ascertainment. *Am J Obstet Gynecol MFM*. 2023;5(2):100814. doi:10.1016/j.ajogmf.2022.100814

10. Labgold K, Stanhope KK, Joseph NT, Platner M, Jamieson DJ, Boulet SL. Validation of Hypertensive Disorders During Pregnancy: ICD-10 Codes in a High-burden Southeastern United States Hospital. *Epidemiol Camb Mass*. 2021;32(4):591-597. doi:10.1097/EDE.0000000000001343

11. Chu YT, Ng YY, Wu SC. Comparison of different comorbidity measures for use with administrative data in predicting short- and long-term mortality. *BMC Health Serv Res*. 2010;10:140-140. doi:10.1186/1472-6963-10-140

12. Quan H, Sundararajan V, Halfon P, et al. Coding algorithms for defining comorbidities in ICD-9-CM and ICD-10 administrative data. *Med Care*. Published online 2005:1130-1139.

13. HCUP CCS. HCUP CCS-Services and Procedures. Healthcare Cost and Utilization Project (HCUP). May 2021. Agency for Healthcare Research and Quality, Rockville, MD. Published May 26, 2021. Accessed November 28, 2023. https://hcup-us.ahrq.gov/toolssoftware/ccs_svcsproc/ccssvcproc.jsp

14. Tepper NK, Boulet SL, Whiteman MK, et al. Postpartum venous thromboembolism: incidence and risk factors. *Obstet Gynecol*. 2014;123(5):987-996. doi:10.1097/AOG.0000000000000230

© 2024 Agarwal A et al. *JAMA Network Open.*
