# Supplementary material for: Health Service Use and Costs During Pregnancy Among Privately Insured Individuals With Congenital Heart Disease
Source: JAMA Netw Open. 2024 May 13;7(5):e2410763. doi: 10.1001/jamanetworkopen.2024.10763 (PMC11091763; doi:10.1001/jamanetworkopen.2024.10763)
Supplement: Supplement 2. — Data Sharing Statement [file jamanetwopen-e2410763-s002.pdf]

Data Sharing Statement

Agarwal. Health Service Use and Costs During Pregnancy Among Privately Insured Individuals with Congenital Heart Disease. *JAMA Netw Open*. Published May 13, 2024. doi:10.1001/jamanetworkopen.2024.10763

Data
Data available: No

Additional Information
Explanation for why data not available: We have deidentified data from claims database and will need permission from them before considering sharing it
